# Semi-Automatic Segmentation of Vertebral Bodies in MR Images of Human Lumbar Spines

**DOI:** 10.3390/app8091586

**Published:** 2018-09-07

**Authors:** Sewon Kim, Won C. Bae, Koichi Masuda, Christine B. Chung, Dosik Hwang

**Affiliations:** 1 School of Electrical Engineering, Yonsei University, Seoul 06974, Korea; 2 Department of Radiology, VA San Diego Healthcare System, San Diego, CA 92161-0114, USA; 3 Department of Radiology, University of California-San Diego, La Jolla, CA 92093-0997, USA; 4 Department of Orthopedic Surgery, University of California-San Diego, La Jolla, CA 92037, USA

**Keywords:** semi-automatic segmentation, MR spine image, vertebral body, graph-based segmentation, correlation

## Abstract

We propose a semi-automatic algorithm for the segmentation of vertebral bodies in magnetic resonance (MR) images of the human lumbar spine. Quantitative analysis of spine MR images often necessitate segmentation of the image into specific regions representing anatomic structures of interest. Existing algorithms for vertebral body segmentation require heavy inputs from the user, which is a disadvantage. For example, the user needs to define individual regions of interest (ROIs) for each vertebral body, and specify parameters for the segmentation algorithm. To overcome these drawbacks, we developed a semi-automatic algorithm that considerably reduces the need for user inputs. First, we simplified the ROI placement procedure by reducing the requirement to only one ROI, which includes a vertebral body; subsequently, a correlation algorithm is used to identify the remaining vertebral bodies and to automatically detect the ROIs. Second, the detected ROIs are adjusted to facilitate the subsequent segmentation process. Third, the segmentation is performed via graph-based and line-based segmentation algorithms. We tested our algorithm on sagittal MR images of the lumbar spine and achieved a 90% dice similarity coefficient, when compared with manual segmentation. Our new semi-automatic method significantly reduces the user’s role while achieving good segmentation accuracy.

## 1. Introduction

**Figure 1.**
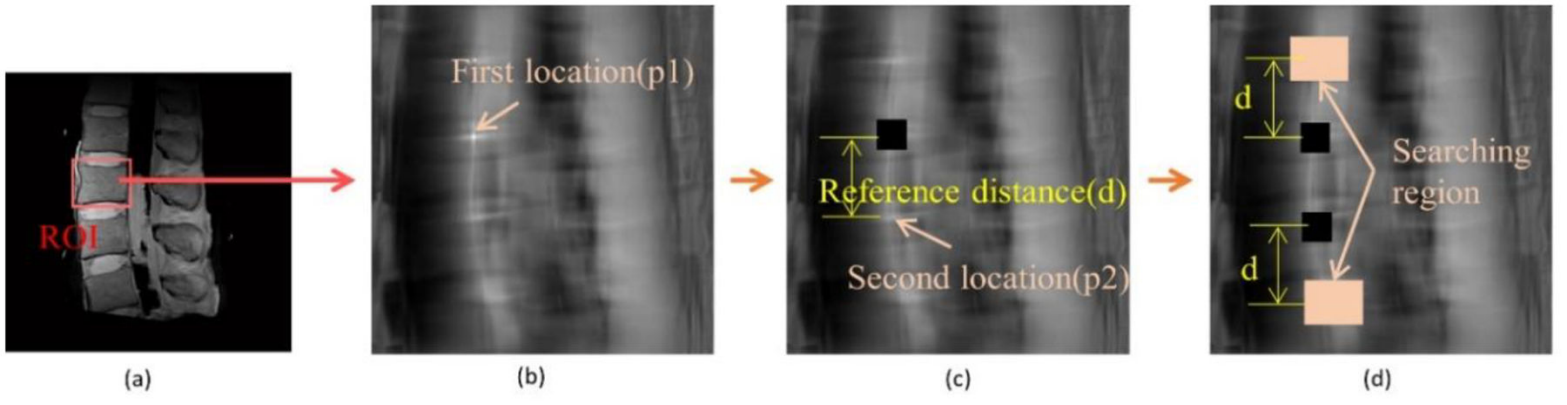
Protocol to find vertebral body ROIs using minimum user input. (**a**) T2-weighted spin echo MR image of a cadaveric lumbar spine and a user-defined ROI placement. (**b**) Correlation map and the location of the user-defined ROI (the first location, p1 by the maximum value). (**c**) Determination of the second location, p2, of the next closest vertebral body and the reference distance, d. (**d**) Detection of the other remaining ROI locations utilizing the reference distance, p1, and p2. Black pixels in (**c**) and (**d**) are disregarded pixels for the next ROI search.

Low back pain is a common disease in modern society [1,2]. As a multifactorial disease, numerous lumbar components including intervertebral discs, paraspinal muscle, or alterations of the vertebral body may contribute to low back pain. Magnetic resonance (MR) imaging is a noninvasive imaging modality that is widely used for both morphological and quantitative evaluation of the human lumbar spine. Evaluation of the vertebral bodies in MR images (Figure 1a) plays a key role in the diagnosis and establishing treatment strategies. Vertebral body segmentation on MR images provides clinically useful information including quantitative biomarkers, volume, and shape. Previous methods for vertebral body segmentation are inherently challenging, owing to the similar signal intensity of the vertebral body and anatomically contiguous tissues and the inconsistent boundaries [3,4].

The segmentation of grayscale medical images has been extensively researched [5]. Some of the classical methods are described here. Histogram-based segmentation is one of the most commonly used methods [6,7]; it uses only pixel intensity to segment the image by applying a threshold. However, this method faces many limitations, because it does not include shape or position information. Another conventional method is the region-growing method that begins by placing a seed [8–11]. This seed point grows on the basis of the similarity of its neighboring pixels and extends out to fill a region of interest (ROI). This method is also based on pixel intensity, which is disadvantageous in the case of noisy images. Another approach employs an edge detection algorithm. For example, the Canny edge-detection algorithm obtains vertical and horizontal gradients using various kernels after limitation of noise via medial or Gaussian filters [12]. Subsequently, non-maximum suppression is used to make the edge thinner and more precise, whereas thresholding and edge tracking further improves the edge. Clustering is another commonly used method. The K-means clustering algorithm determines the number of clusters, k, with the initial center position of the clusters and repeats the process until the center position of the clusters and their corresponding data converge [13–15]. The fuzzy C-means algorithm is another clustering method that sets the number of clusters and the objective function with initial values and repeats the calculation until the objective function is minimized [16–19].

The actively investigated segmentation method in recent medical imaging is the graph-cut algorithm. This algorithm transforms an image into a graph G = (V, E), where V (node) is the actual spatial element to be segmented and E (edge) is the similarity of each pixel [20–24]. Depending on how V and E are specified, various graphs can be created. The generated graph is transformed into a similarity matrix to perform division. Various algorithms, such as the min-cut max-flow algorithm, are used for the division [25,26]. Use of the graph-cut algorithm for medical image segmentation has shown good results. Egger and Kapur reported a 91% Dice similarity coefficient (DSC) [27], whereas Schwarzenberg and Freisleben reported an 81% DSC using the graph-cut method for vertebral body segmentation [3]. However, in both these studies, each vertebral body was individually segmented, and the method required repeated user inputs to assign a seed point at the center of each vertebral body. Furthermore, because the performance of the algorithm depends on the number of nodes and the connection method, the method requires the user to calibrate multiple parameters for each segmentation.

The most recent approaches are the deep-learning-based methods, which exhibit superior performance over existing mathematical algorithms. Ronneberger reported successful segmentation of medical images using a U-net image segmentation network [28]. Korez also showed the possibility of a deep learning segmentation for vertebral bodies in MR lumbar spine images [29]. However, these deep neural networks require a large amount of data and use a large amount of memory, which is a key limitation. For example, it might be difficult for a single graphics processing unit (GPU), sold in the market, to learn and use the U-net with a 512 × 512 size image matrix. Furthermore, a repeated heavy training processes may be required for the segmentation of different types of images (e.g., use of different contrasts, sequences, or different MR scanners). On the other hand, mathematical algorithms do not require a large amount of training datasets and can be applied to large sized images. Unlike common optical images, the acquisition of a large amount of data is a key limitation in medical imaging. Therefore, the segmentation methods that employ mathematical algorithms for medical image processing are still needed.

Our proposed method aims to reduce user inputs to a minimum while still achieving high accuracy in a short execution time during the segmentation of vertebral bodies. We first reduce the number of user inputs for vertebral body selection (i.e., a rectangular ROI containing a single vertebral body) to just one for the whole image. The remaining vertebral bodies are automatically detected using a correlation algorithm without any further user inputs. Subsequently, the boundary of the vertebral body inside each ROI is automatically segmented using the graph-cut and line-based methods together with the incorporation of the Hough transform and edge-detection algorithm. No additional user input is required. A preliminary study of this paper was partially presented at the annual meeting of International Conference on Electronics, Information, and Communication in 2017 [36].

This paper comprises four sections. Section 2 describes the details of our proposed algorithm. Section 3 presents the experimental results. Finally, Section 4 discusses the study as a whole and concludes the article.

## 2. Materials and Methods

### 2.1. Materials

This cadaveric study was exempted from institutional review board approval. Nineteen lumbar spines (L1 through L5) from cadaveric donors (age range 46–60 years) were obtained from a local tissue bank. MR imaging was performed on a 3-Tesla system (General Electric Signa HDx, San Diego, CA, USA). The cadaveric spines were placed in the supine position to ensure vertical alignment of the vertebral bodies in the MR images. Our segmentation algorithm was developed on the basis of this position, which is typically used in many MR imaging protocols. A T2-weighted fast spin echo sequence was used with the following parameters: Mid-sagittal plane; repetition time (TR) = 2000 ms; echo time (TE) = 7.6 ms; field of view (FOV) = 180 − 220 mm; pixel spacing= 0.39 mm; slice thickness = 3 mm; flip angle= 90°; and bandwidth = ±62.5 kHz. Image processing and test experiments were conducted using Matlab R2012b (The Mathworks Inc., Natick, MA, USA).

### 2.2. ROI Detection

The first part of our algorithm requires minimal user inputs regarding the location of one of the vertebral bodies and the size of the rectangular ROI window (width: lx and height: ly); the automated algorithm then searches other locations with the same window for the remaining vertebral bodies. This ROI detection step is necessary to increase the efficiency of the subsequent segmentation process by setting uniform ROIs, each of which contains only one vertebral body. When the user defines a rectangular area containing a single vertebral body along with some of the surrounding tissues, as shown in Figure 1a, the correlation algorithm [30] obtains a correlation map (Figure 1b) between the user-defined ROI and other candidate ROIs throughout the image.

When examining the correlation map (Figure 1b), the highest correlation value is easily found at the center position (p1) of the user specified ROI. The next vertebral body location is searched by finding the second highest correlation value. However, the pixels in the immediate vicinity of p1 tend to have high correlation values that are close to the correlation value of p1. Therefore, correlation values in the immediate neighborhood of p1 (equivalent to 1/3 of the ROI size) are disregarded. Then, the search continues for the next highest correlation value and the corresponding location of the next vertebral body, p2. After finding p2, the neighborhood of p2 in the correlation map is also disregarded to search for the next vertebral body locations. In this case, we use a reference distance, d, defined as the distance between p1 and p2 (Figure 1c) for subsequent searches. The searching area can be reduced to the small area whose width and height are lx and ly, respectively, because the reference distance provides the approximate locations of the upper or lower vertebral bodies. Through this process, vertebral body positions are found in the entire image and each ROI for the individual vertebral body is set for subsequent fine segmentation, as described in the next section.

The information on the intervertebral disc can also be used to further refine the vertebral body location. The searched locations are corrected again using these intervertebral discs that are sandwiched between the vertebral bodies. Assuming that an intervertebral disc ROI exists in the lx/2 portion just above the vertebral body ROI, locations of other intervertebral discs can also be obtained in the same manner (Figure 1). The horizontal locations of the vertebral bodies and the intervertebral discs are generally similar. Therefore, if the horizontal location of a searched vertebral body ROI is significantly different from the horizontal location of the surrounding intervertebral disc, the horizontal location of a searched vertebral body ROI needs to be adjusted. When the difference between the horizontal locations of the searched vertebral body and the intervertebral disc is larger than 10% of ly, the horizontal location of the searched vertebral body ROI is replaced with that of the searched intervertebral disc ROI.

### 2.3. Roi Fine Tuning—Hough Transform and Canny Edge Filtering

The vertebral bodies are slightly different in size and these may be rotated with respect to each other due to kyphosis or lordosis [31]. Therefore, after identifying ROIs for the vertebral bodies, the ROIs need to be fine-tuned to make the orientation and size of the vertebral bodies similar to each other for ease of segmentation. Canny edge filtering and the Hough transform [12,32] are used for this purpose.

#### 2.3.1. ROI Fine Tuning—Orientation Adjustment

**Figure 2.**
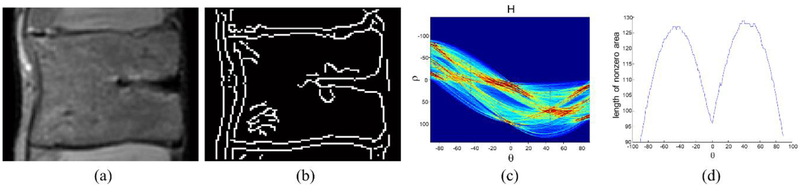
Analysis for the ROI refinement of the vertebral body. (**a**) Vertebral body ROI. (**b**) Edge of the vertebral body ROI. (**c**) Hough transformed image of the vertebral body ROI. (**d**) Length of nonzero area at each angle.

First, Canny edge filtering is applied to the vertebral body region **R** to extract the edge components **E** of the region. Second, a Hough transform is applied to **E** to estimate the rotation angle of the vertebral body. **H** is the Hough transformed image of **E**, as shown in Figure 2c. The x-axis of **H** is a rotation angle, and the y-axis of **H** is the distance from origin. Assuming that the shape of the vertebral body is similar to a rectangle, the y-axis value of **H** has the smallest value at 0°. However, when the vertebral body rotates, the x-coordinates of the smallest **H** deviates from 0°; the degree of deviation indicates the rotation angle of the vertebral body. Therefore, the ROI can be adjusted by rotating it in the direction opposite to that of the estimated rotation angle.

#### 2.3.2. ROI fine Tuning—Boundary Adjustment

Define **Px**(x) as the result of projecting **E** on the x-axis, and **Py**(x) as the result of projecting **E** on the y-axis. lx and ly represent the size of the vertebral body ROI. As the vertebral body is rectangular, the minimum values of **Px** and **Py** are $Px\left(\frac{lx}{2}\right)$ and $Py\left(\frac{ly}{2}\right)$. On the other hand, the projection of the left and right sides of the vertebral body edge will be at both ends of **Px**, having considerably larger values than $Px\left(\frac{lx}{2}\right)$. Similarly, the point at which the upper and lower sides of the vertebral body edge are projected will be at both ends of **Py**, having larger values than $Py\left(\frac{ly}{2}\right)$. Therefore, if $max(Px\left(2<i<\frac{lx}{2}\right)<\alpha Px\left(\frac{lx}{2}\right))$, it implies that the ROI does not include the left side of the vertebral body, and hence, the ROI should extend to the left. Likewise, if $max(Px\left(\frac{lx}{2}<i\leq lx-2\right))<\alpha Px\left(\frac{lx}{2}\right)$, the ROI should extend to the right. This rule is also applied to the relationships between **Py** and both the upper and lower sides of the ROI, such that the ROI can contain the vertebral body within a specified interval. This process allows us to adjust vertebral body ROIs so that they contain all the necessary boundaries of the vertebral body, thereby increasing the effectiveness of the subsequent segmentation.

### 2.4. Segmentation

**Figure 3.**
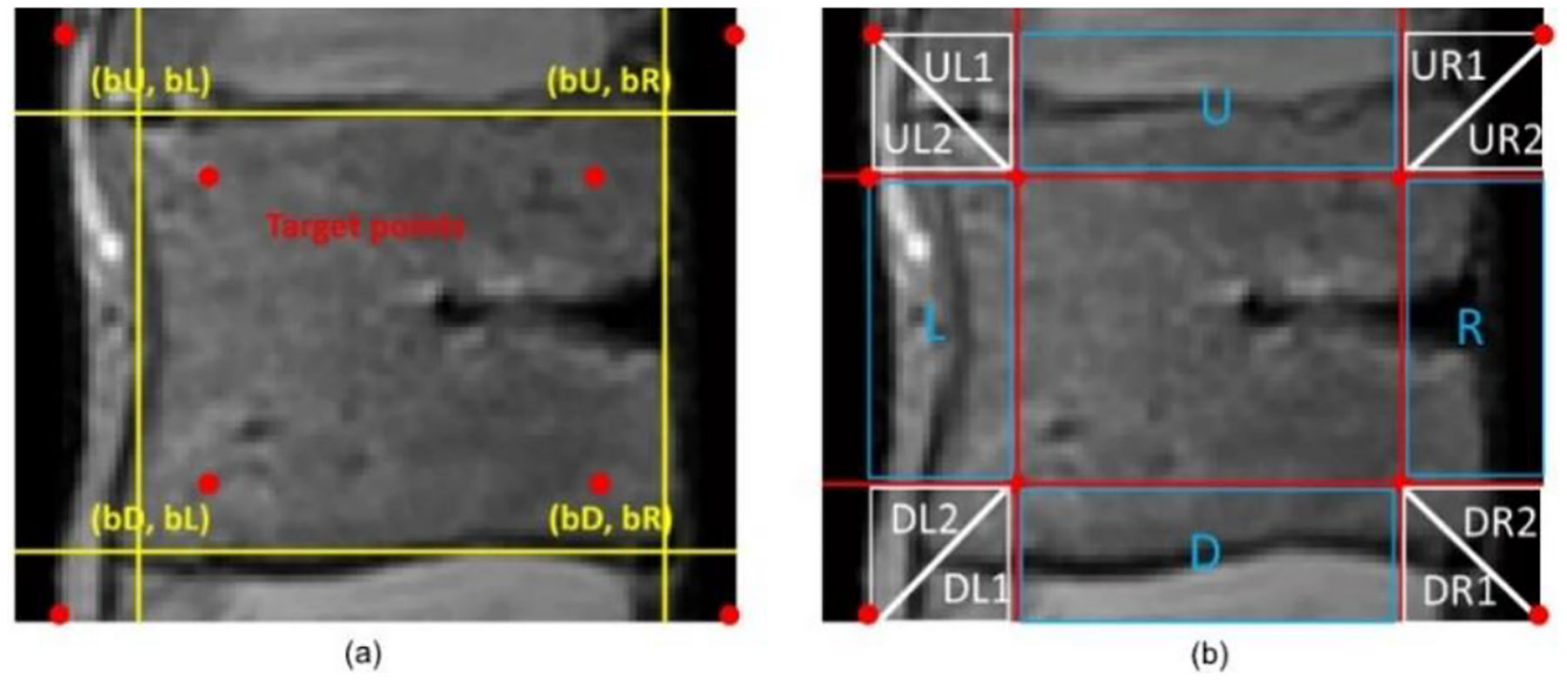
Partitioning area. (**a**) Pointing base vertices. (**b**) Partitioned area.

**Figure 4.**
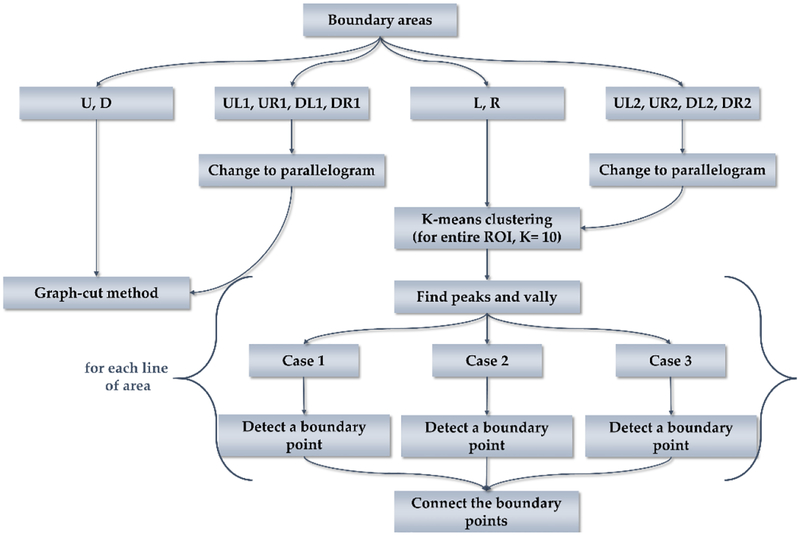
Flow chart of segmentation process.

During the ROI fine tuning process, we can determine the approximate position of the vertebral body boundary.

(1) $$bL=\underset{i}{argmax}\left(Px\left(1<i<\frac{lx}{2}\right)\right)$$

(2) $$bR=\underset{i}{argmax}\left(Px\left(\frac{lx}{2}<i\leq lx\right)\right)$$

(3) $$bU=\underset{i}{argmax}\left(Py\left(1<j<\frac{ly}{2}\right)\right)$$

(4) $$bD=\underset{i}{argmax}\left(Py\left(\frac{ly}{2}<j\leq ly\right)\right)$$

where (bL, bU), (bR, bU), (bL, bD), and (bR, bD) are approximate vertices of the vertebral body boundary. Then, we draw lines connecting each dot at points spaced inward and outward from the approximate vertices, as shown in Figure 3a. Using these points, the vertebral body ROI is divided into eight areas, as illustrated in Figure 3b, and different segmentation methods are applied to these areas. Figure 4 shows the flowchart of the segmentation process. Areas **U** and **D** detect boundaries using the graph-cut based method. **U** and **D** are converted to graph **G** = (**V**,**E**). As **G** should be divided into the top and bottom, the nodes n **V** and edges e ∈ **E** are set along the x-axis. Intervals between each pixel along the x-axis are set to the nodes n ∈ **V** and pixel intensities between the nodes are set to the edges e ∈ **E** [25]. As a result, boundaries that can properly distinguish the top and bottom in the x-direction of **U** and **D**are detected.

Because **L** and **R** contain the background, applying the graph-cut to find the minimum route is difficult. In this case, segmentation is performed by capturing boundary points for each line of the ROI. First, k-means clustering is performed to divide the total ROI into 10 clusters. Let **YL** be the clustered **L**, whose size is (lx, ly) and **YL**_**i**_ be the i-th line of **YL**. At this time, the base value BL_i_ is ly or the peak point nearest to ly, and the reference value RL_i_ = *ceil*(**YL**_**i**_(BL_i_)/2). **YL**_**i**_ can be divided into three cases. Case 1 has one or more deep valleys and peaks between BL_i_ and 1, and the peak value far from BL_i_ is larger than RL_i_. Case 2 is similar to the case 1, but the peak values are smaller than RL_i_. Case 3 is the case without a valley. For each case, the boundary point EL_i_ is as follows:

(5) $$Case\;1:\;\;{EL}_{i}\;=\underset{j}{argmin}\left({YL}_{i}\left({PL}_{i}<j<ly\right)\right)$$

(6) $$Case\;2,3:\;\;{EL}_{i}\;=\;{ML}_{i}$$

PL_i_ denotes the peak point closest to BL_i_ which has a valley smaller than RL_i_ towards BL_i_ and having a larger value than the valley by 2 cluster stages or more. ML_i_ is the point closest to BL_i_ among the points having the value of *ceil*(**YL**_**i**_(BL_i_)/3). The case of region **R** is similar to the case of **L**, but performed in the opposite direction. **YR** is the clustered **R**, whose size is (rx, ry) and let **YR**_**i**_ be the i-th line of **YR**. BR_i_ is 1 or the peak point nearest to 1, and the reference value RR_i_ = *ceil*(**YR**_**i**_(BR_i_)/2). Case 1 has one or more deep valleys and peaks between BR_i_ and ry, and the peak value far from BR_i_ is larger than RR_i_. Case 2 is similar to case 1, but the peak values are smaller than RR_i_. Case 3 is the case without a valley. For each case, the boundary point ER_i_ is as follows:

(7) $$Case\;1:\;\;{ER}_{i}=\;\underset{j}{argmin}\left({YR}_{i}\left(1<j<{PR}_{i}\right)\right)$$

(8) $$Case\;2,3:\;\;{ER}_{i}\;=\;{MR}_{i}$$

PR_i_ denotes the peak point closest to BR_i_, which has a valley smaller than RR_i_ towards BR_i_ and having larger value than the valley by 2 cluster stages or more. MR_i_ is the point closest to BR_i_ among the points having the value of *ceil*(**YR**_**i**_(BR_i_)/3).

Before the segment the **UL1**, **UL2**, **UR1**, **UR2**, **DL1**, **DL2**, **DR1**, and **DR2** have to change their triangular shape to parallelograms by copying points. Subsequently, the segmentation method of **U** and **D** is applied to **UL1**, **UR1**, **DL1**, and **DR1** areas, and the segmentation method of **L** and **R** is applied to the **UL2**, **UR2**, **DL2**, and **DR2** areas.

Finally, when all edge points are found, the cubic function is fitted for each side of the boundary to remove the edge points above the error range. The points that deviate by a regular distance from the cubic function curve are replaced with points on the function curves, and finally the segmentation process is ended by connecting all the points.

## 3. Results and Discussions

### 3.1. ROI Detection

**Figure 5.**
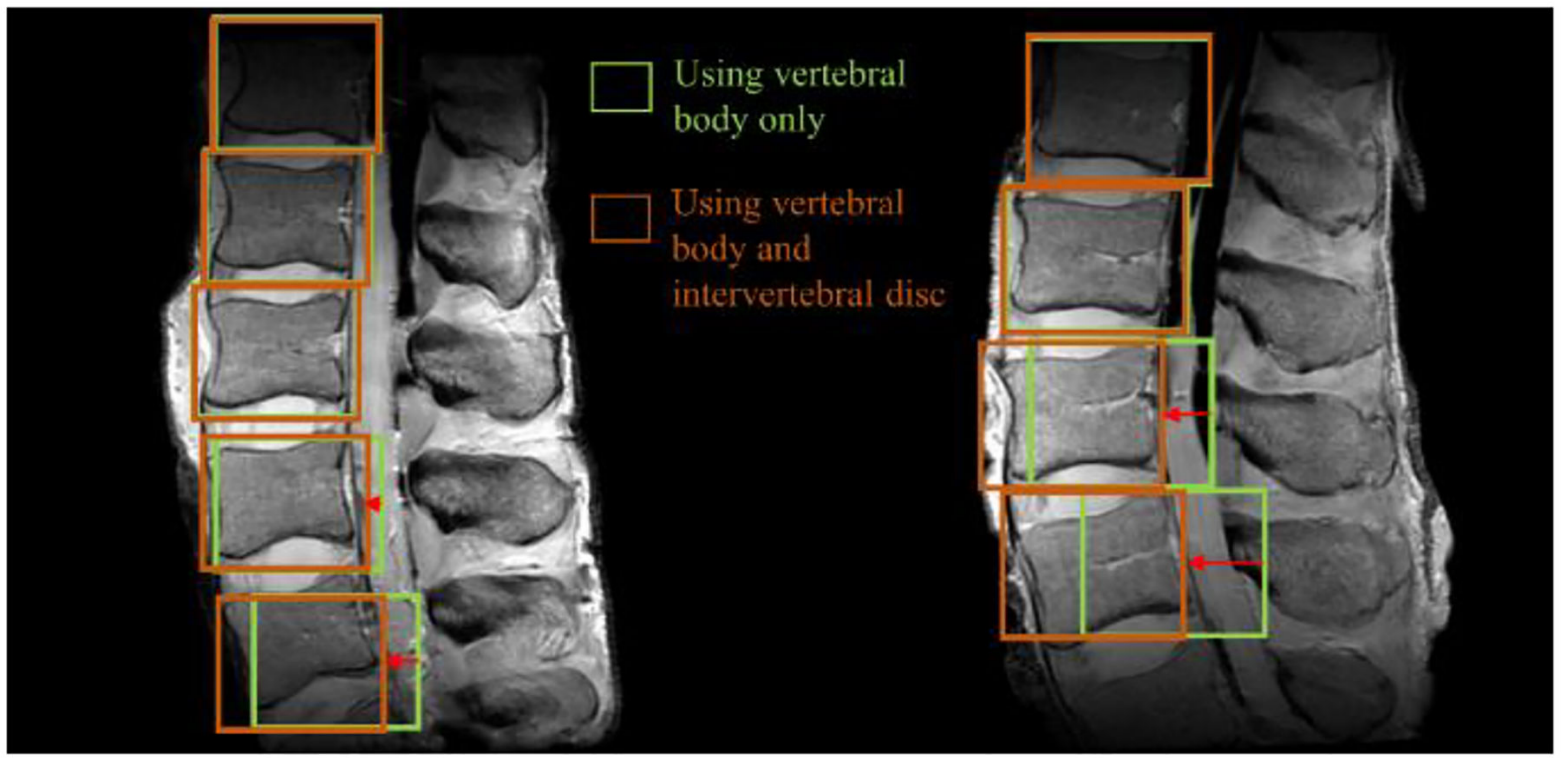
Results of the ROI detection. Green boxes are the results of using only vertebral body ROI, and brown boxes are the results of using both vertebral body ROI and intervertebral disc ROI.

We tested the ROI detection algorithm using the whole image and user-defined ROI as the minimum user input. The first test was performed with only vertebral body correlation maps, and the second test was performed with both correlation maps of the vertebral body and intervertebral disc. For all 85 vertebral bodies in 19 MR spine images, the number of detection failure cases in the vertebral body test was three, whereas the vertebral body and intervertebral disc test was zero. Detection error of the vertebral body and the intervertebral disc test was 10.41 pixels, which was 19.36% lower than that of the vertebral body test (12.91 pixels). Therefore, it is clear that the use of both ROIs yields better results and that the intervertebral disc exhibits more precise information of location as compared with the vertebral body. Figure 5 also shows that the use of the intervertebral discs adjusts the ROI detection more closely. Detection of ROIs using the vertebral body ROI offered by the user poses a difficulty only in finding other vertebral bodies of the MR spine images; however, more precise detection can be performed if the intervertebral disc ROI is additionally used for ROI detection. In addition, when the user ROI was set to the central vertebral body, there was no detection failure case. On the other hand, when the ROI was set to the uppermost vertebral body, the detection failure case occurred 0.93 times per image, and when the ROI was set to the undermost vertebral body, the detection failure case occurred 0.77 times per image.

In most cases, the vertebral bodies and intervertebral discs are at a similar position in the horizontal direction. In other words, knowing the position of the intervertebral discs helps to understand the horizontal position of the vertebral bodies. As the intervertebral discs have more compact and unique shapes than the vertebral body, their correlation with other tissues is quite small. Therefore, more accurate locations of vertebral bodies can be found using correlation maps of the intervertebral discs.

Aligning the ROIs with a fixed size cannot completely cover all the vertebral bodies because the vertebral bodies have various sizes and shapes. The ROI fine tuning process adjusts the ROIs to be fitted for each of these various vertebral bodies and aligns them to the center of the ROIs. When the ROI fine tuning process was not applied, there were several cases in which a part of the vertebral body was not included within the ROI, on average, of 1.31 cases per image.

### 3.2. Segmentation Results

Segmentation experiments were performed on 19 MR spine images including 85 vertebral bodies. All user ROIs were manually specified vertebral body regions. The DSC was used to evaluate the accuracy of segmentation, and the references were manually obtained by an expert [33]. DSC is defined as

(9) $$DSC=\;\frac{2\left|F_{manual}\cap F_{result}\right|}{\left|F_{manual}\right|+\;\left|F_{result}\right|}$$

where **F**_manual_ represents the manual references and **F**_result_ represents the segmentation results of the proposed method. Table 1 shows the DSC results for 19 MR spine images.

The average DSC was 89.91% (Table 1). This is a valuable result as it is comparable to the 90.97% achieved by Egger et al. who used the square-cut algorithm that requires more user inputs than the proposed method [27]. Further, it is higher than the 81.33% reported by Schwarzenberg et al. who used the cube-cut algorithm [3]. The standard deviation score 2.77% is similar to that reported by Egger et al. (2.2%) [27] and much smaller than that reported by Schwarzenberg et al. (5.07%) [3]. For the number of voxels and the vertebral body volume, the square-cut algorithm showed a difference of 3.83% on both, compared with manual segmentation results [27], and the cube-cut algorithm showed differences of 15.95% and 5.97%, respectively, compared with manual segmentation results [3]. Our method, on the other hand, showed differences of 3.05% and 2.78% respectively, compared with manual segmentation results. Our results are lower than the cube-cut algorithm and square-cut algorithm. While a direct comparison of the results obtained with different algorithms is difficult, owing to differences with respect to image contrast type, resolution, image quality, and the number of test datasets used in each study, it generally suggests a comparable performance range of our algorithm. The square-cut and cube-cut algorithms, which are based on the graph-cut method, have no ROI detection processes; therefore, these algorithms require the locations of all vertebral bodies. Furthermore, because the results depend on the cost of constructing the graph, these algorithms require users to adjust the parameters such as the number of nodes and edges according to the circumstances. On the other hand, the proposed method requires the user to draw just one vertebral body ROI. Table 2 shows the comparison of the user inputs between Square-cut and Cube-cut algorithms and our method. Square-cut and Cube-cut algorithms requires four parameters to adjust and seed points for all vertebral bodies, but our method only needs a single vertebral body ROI. This suggests that our method can maintain or improve the segmentation performance despite reduction in the number of user inputs, and the automated portion of the whole segmentation process increases, as compared with the existing methods.

**Figure 6.**
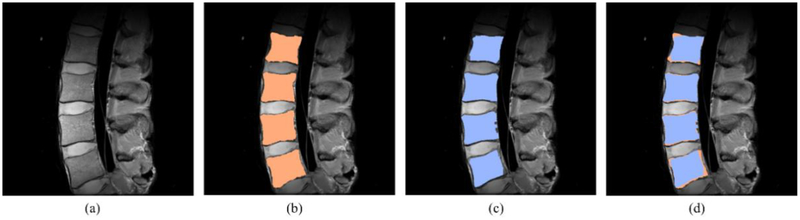
Result of the segmentation algorithm. (**a**) Original image. (**b**) Manual segmentation result. (**c**) The result of the proposed segmentation algorithm. (**d**) The comparison between (**b**) and (**c**).

Figure 6 shows a representative segmentation result, wherein Figure 6b shows the manually segmented areas, and Figure 6c shows the results of the proposed method. Figure 6d shows the difference between the manual references and the result of the proposed method. The results of the proposed method are generally similar with the manual references. It can be seen that four vertebral bodies are correctly identified and segmented and that most regions are recognized as vertebral bodies. Nonetheless, there are some limitations. The results of the proposed method have non-smooth boundaries, as compared with the manual references. When examining each vertebral body, the top and bottom boundaries are smooth and match well with the actual reference, whereas the left and right boundaries are not entirely smooth and have protruding parts. Based on these experiments and evaluations, it seems that the results of the line-based algorithm are not as good as those obtained with the graph-based algorithm.

**Figure 7.**
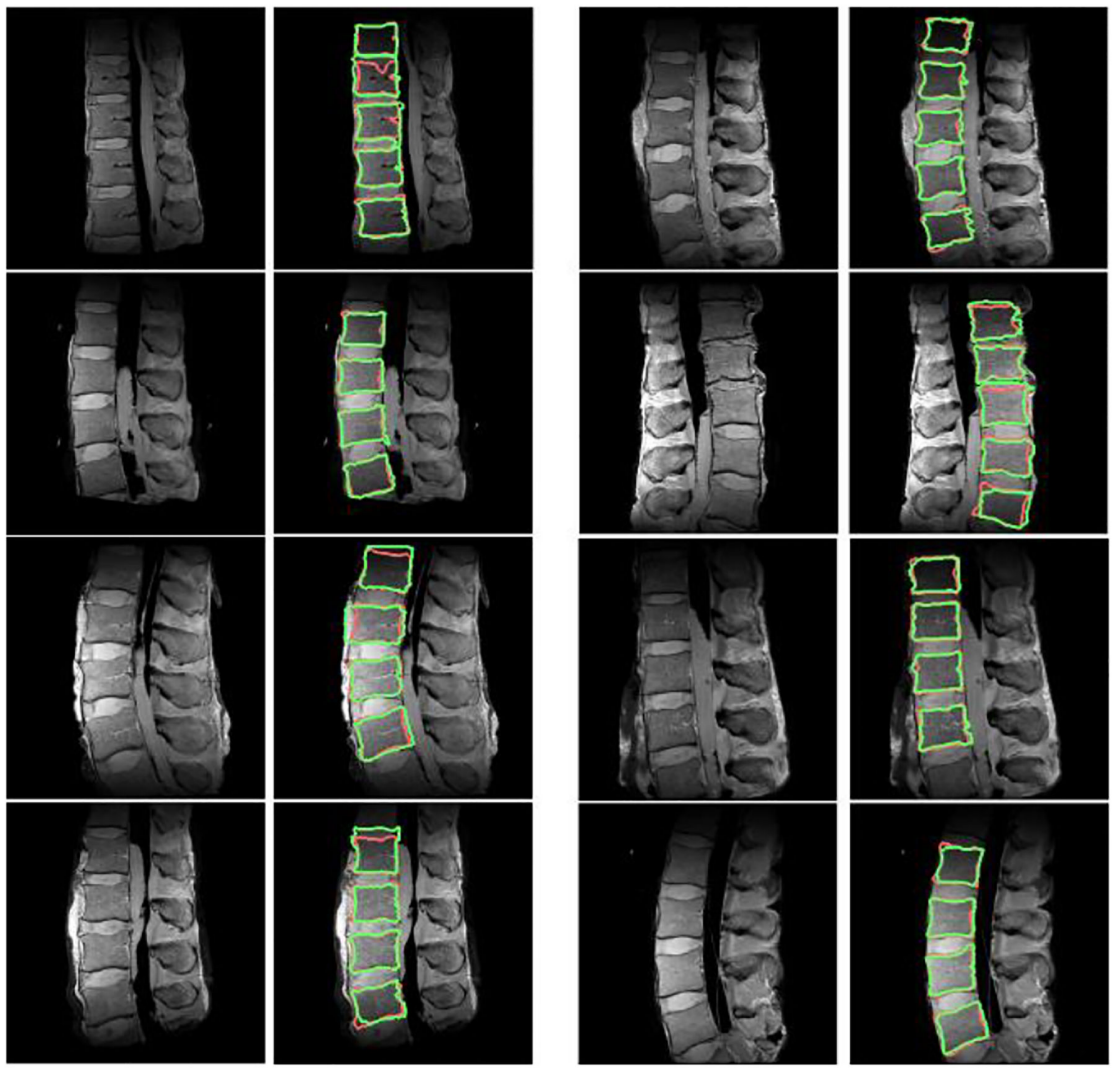
Segmentation results for other magnetic resonance (MR) spine images. Manual segmentation results (red line). Simulation results (green line).

Figure 7 shows eight different examples of the results of the proposed method. It can be observed that the overall performance looks good in terms of ROI detection and the subsequent segmentation has an average DSC of 89.91 (Table 1). These results show that the proposed method has successfully reduced the user’s role while maintaining a performance comparable to existing methods. There are slight differences between the manual references and the results of the proposed method with respect to the upper and lower parts of the images. The proposed method performs segmentation depending on the relative contrast of pixel intensities in the ROI. Therefore, the proposed method may not be able to achieve accurate segmentation if the contrast of the images is not good as shown in the upper and lower parts of the images in Figure 7. This problem may arise with most segmentation algorithms depending on the image contrast.

For additional experiments, the data augmentation technique using a rotation process was applied to the original sample images. 19 MR images were rotated by 10°, 20°, and 30° with respect to the vertical axis. Therefore, a total of 51 rotated images were obtained. Table 3 shows the segmentation results for them. The average DSC scores were 89–90 in all the cases, and the standard deviations of the DSC scores were about three, which was similar to the results of the original sample MR images. Both results in Table 1 and Table 3 demonstrate the robustness of our method.

## 4. Conclusions

Obtaining information about the vertebral bodies in the lumbar spine is very important for the diagnosis and treatment of low back pain [34]. In this study, we proposed a semi-automatic algorithm for vertebral body segmentation that requires the user to specify only a single ROI. Our method overcomes the disadvantages of the existing algorithms that require multiple user inputs, such as clicking on each vertebral body, assigning ROIs for each vertebral body, or adjusting multiple parameters depending on circumstances. In our method, only one user-defined-ROI is required to identify the ROIs for all vertebral bodies in the image. Additionally, the detected ROIs were finely adjusted using the edge-detection algorithm and Hough transformation to consider the orientation and size of the vertebral bodies. The subsequent automatic segmentation was performed by combining both the line-based and graph-cut-based methods depending on the parts of the bodies. The experimental results demonstrate comparable or an even higher performance than the existing methods, even though the automated portion of the entire segmentation process is increased. Furthermore, our method does not require a heavy training dataset necessary for deep-learning-based methods and can be applied regardless of the image size.

In future studies, we will apply this technique to in vivo datasets and evaluate its performance for various types of contrast images obtained from living patients. The in vivo datasets may have different noise levels, field of views, contrasts, and resolutions. Motion-related artifacts and abnormality of the vertebral bodies and discs with spinal injuries or diseases should be further investigated. The application of dorsal vertebrae, which was not included in this study, can be a good future topic to expand and evaluate our technique. A three-dimensional (3D) extension of this technique is also one of our future studies. 3D extension of other approaches has yielded better results than those obtained via two-dimensional (2D) methods [28,35]. Our 3D extension is also expected to improve the overall segmentation performance.

## Figures and Tables

**Table 1.** DSC results of the experiments.

No.	Volume of Vertebral Body (mm^3^)		Number of Voxels		DSC (%)
	The Proposed	Manual	The Proposed	Manual	
1	2347.58	2134.11	5723.00	5379.40	90.96
2	2069.50	2059.26	5298.25	5265.50	94.11
3	2904.11	2731.39	7435.00	6882.25	90.50
4	1363.59	1468.81	3491.00	4037.00	88.11
5	2028.70	2034.23	5193.80	5263.20	93.77
6	2422.79	2360.53	6202.75	6003.50	91.01
7	1858.32	1843.76	4757.60	4864.00	86.94
8	2247.85	2136.49	5231.20	4847.00	87.79
9	1762.97	1520.00	4102.80	3548.40	86.58
10	1658.64	1686.57	3860.00	3933.60	85.30
11	2412.74	2172.44	6177.00	5408.00	88.82
12	2376.49	2240.58	6084.20	5695.20	90.20
13	2148.50	2127.33	5500.50	5432.75	92.80
14	3222.66	2872.19	8689.50	7508.25	86.43
15	2667.37	2445.22	7192.25	6443.50	88.81
16	2264.05	2365.08	6104.75	6445.25	93.33
17	2212.97	2296.04	5967.00	6247.00	93.51
18	2494.64	2662.20	6726.50	7231.25	91.34
19	2385.87	2425.71	6433.20	6694.60	87.92
μ ± σ	2255.23 ± 429.94	2188.52 ± 377.83	5798.44 ± 1265.49	5641.56 ± 1110.66	89.91 ± 2.77

**Table 2.** Comparisons of the user inputs between existing algorithms and the proposed method

	Square-Cut & Cube-Cut	The Proposed Method
Parameter tuning	The number of rays	None
	The number of nodes	
	Maximum length of the rays	
	Delta value	
Necessary inputs	Seed points for each vertebral body	A single vertebral body ROI

**Table 3.** Dice similarity coefficient (DSC) results of the additional experiments.

	Images Rotated by 10°	Images Rotated by 20°	Images Rotated by 30°
μ	89.15	89.70	89.55
σ	3.45	2.96	3.18
